# Protocol: using virus-induced gene silencing to study the arbuscular mycorrhizal symbiosis in *Pisum sativum*

**DOI:** 10.1186/1746-4811-6-28

**Published:** 2010-12-14

**Authors:** Mette Grønlund, Anne Olsen, Elisabeth I Johansen, Iver Jakobsen

**Affiliations:** 1 Biosystems Division, Risø National Laboratory for Sustainable Energy, Technical University of Denmark, P.O. Box 49, DK-4000 Roskilde, Denmark; 2 Department of Genetics and Biotechnology, Faculty of Agricultural Sciences, Aarhus University, Thorvaldsensvej 40, DK-1871 Frederiksberg C, Denmark

## Abstract

Virus-induced gene silencing (VIGS) is an alternative reverse genetics tool for silencing of genes in some plants, which are difficult to transform. The pea early-browning virus (PEBV) has been developed as a VIGS vector and used in pea for functional analysis of several genes. However, the available PEBV-VIGS protocols are inadequate for studying genes involved in the symbiosis with arbuscular mycorrhizal fungi (AMF).

Here we describe a PEBV-VIGS protocol suitable for reverse genetics studies in pea of genes involved in the symbiosis with AMF and show its effectiveness in silencing genes involved in the early and late stages of AMF symbiosis.

## Background

Virus-induced gene silencing (VIGS) exploits a natural defence mechanism in plants against virus infection [1]. Virus replication leads to the formation of double-stranded RNA, which is detected by the plant. This triggers post-transcriptional silencing through the production of short interfering RNAs (siRNAs), which target the viral RNA for degradation [2]. Inserting a fragment of a plant gene into a virus vector will result in a recombinant virus that triggers degradation of both the virus transcript and of homologous endogenous plant RNA sequences. Recently, VIGS vectors have been developed for the legume species *Glycine max, Phaseolus vulgaris *[3,4], *Pisum sativum *[5], *Lathyrus odorata *and *Medicago truncatula *[6].

Legume species are important crops and special in their ability to engage in symbioses with both fungi and bacteria. Like approximately 80% of higher plant species, legumes can form root endosymbiosis with arbuscular mycorrhizal fungi (AMF), whose root-external mycelium scavenges a large soil volume for soil nutrients, in particular phosphate. Nutrients are transferred to the plant via arbuscules in the root cortex by transporters up-regulated or specifically expressed during symbiosis [7]. Moreover, legumes specifically interact with nitrogen-fixing *Rhizobium *bacteria to establish a symbiosis in which atmospheric nitrogen is fixed by the symbiotic form of rhizobia and delivered to the plant partner. *P. sativum *has been used as a model for classical mutant analyses, but it has limitations as a model legume for molecular studies since it has a large genome and is difficult to transform [8]. Functional analysis of the genes involved in the legume-microbe symbioses has therefore been performed mainly in two other model legumes, *M. truncatula *and *Lotus japonicus *[9,10]. The development of VIGS vectors for *P. sativum*, *G. max *and *P. vulgaris *has paved the way for functional analysis of plant symbiosis genes in crop legumes and the transfer of knowledge from model legumes to crop plants. The pea early-browning virus (PEBV) VIGS vector is used for gene silencing in *P. sativum *[5], and we have recently described a PEBV-VIGS protocol to silence pea genes involved in the symbiosis with nitrogen-fixing *Rhizobium *[11].

Here our aim was to develop a "mycorrhiza-PEBV-VIGS protocol", to extend the use of VIGS for functional studies of pea genes involved in the symbiosis with AMF. The requirements for such a protocol are to establish virus infection and generation of siRNA molecules for induction of gene silencing in the pea roots before they become AMF colonized. The silencing potential of the mycorrhiza-PEBV-VIGS protocol was evaluated using two target genes *PsSym19 *and *PsPT4*, with known mutant phenotypes. The protocol produced reproducible silencing of both symbiosis genes at levels which were sufficient to produce symbiotic phenotypes.

### Materials

#### Plants, root symbionts, growth media and growth conditions

The *P. sativum *cultivars 'Dark Skinned Perfection' (Dæhnfeldt, Odense, Denmark) and 'Bilbo' (Toft planteforædling, Roslev, Denmark) were used in the PsSym19 and PsPT4 studies, respectively. Pea seeds were surface-sterilized with 1.5% hypochlorite and pre-germinated for two days in the dark prior to sowing. For the nodulation experiments, *Rhizobium leguminosarum *bv. viceae strain 248 [12] was grown in YMB medium [13] at 28°C with shaking to OD600 = 0.3-0.5 and applied to the pea roots by pipetting 10 ml culture onto the free surface of the nurse pot before watering. For mycorrhiza-experiments the inoculum consisted of a mixture of dry soil, spores and fragments of *Trifolium subterraneum *roots colonised by *Glomus intraradices *BEG87.

Two growth media (GM) were used: "GM-A" was a semisterile (15 kGy, 10 MeV electron beam) 1:1 soil/sand mixture (7 mg P kg^- 1 ^soil;[14]) supplied with nutrients, except P [15] and watered to 60% of the water-holding capacity. "GM-B" was expanded clay (Leca^®^, 2-5 mm; Maxit, Risskov, Denmark) supplied with a low P nutrient solution (2.52 mM CaCl_2_; 1 mM KNO_3_; 2 mM MgSO_4_; 0.083 mM K_2_HPO_4_; 0.017 mM KH_2_PO_4_; 0.3 μM CuSO_4_; 4.6 μM MnSO_4_; 0.8 μM ZnSO_4_; 23.1 μM H_3_BO_3_; 0.2 μM Na_2_MoO_4_; 0.5 μM Fe-EDTA-di-hydroxyphenylacetate; S. Kosuta, personal communication). For both GM-A and GM-B the pea pots were supplied with 50 mg NH_4_NO_3_-N per pot at 2 and 3 weeks after sowing. Plants were grown in climate chambers with a 16/8 hr light-dark cycle at 20/15°C.

#### Statistical analysis

Standard deviations were calculated and pair-wise analysis by Student t-test was performed using calculators provided at http://www.physics.csbsju.edu/stats/.

## Protocol

### Preparation of VIGS constructs

**Figure 1:**
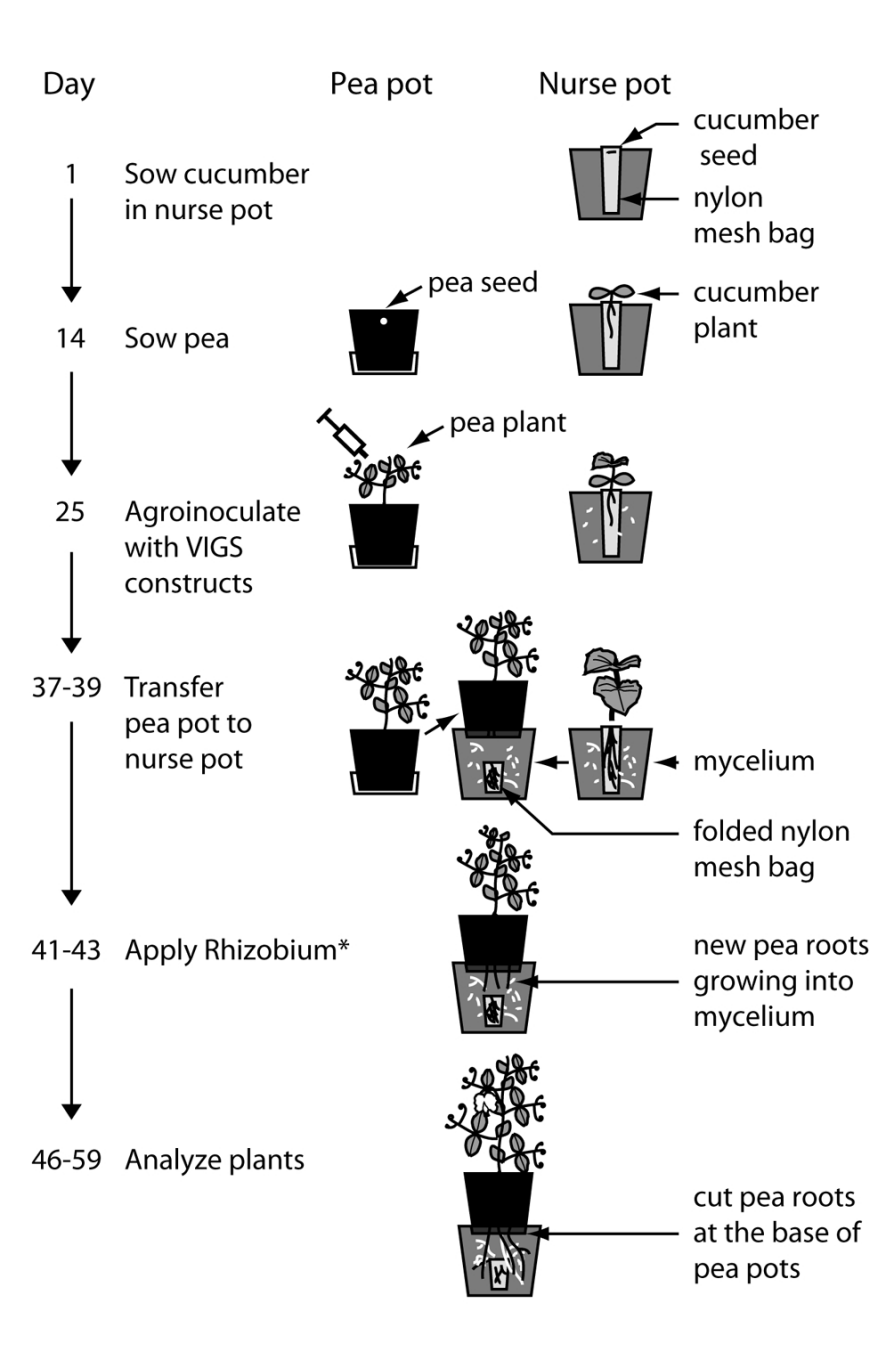
**Protocol for virus-induced gene silencing for functional studies of mycorrhiza-related genes of *Pisum sativum***. Day 1: Sow pre-germinated cucumber seeds (right pot) in nylon mesh bag in the "nurse pots". Day 14: Sow pre-germinated pea seeds (left pot) in the "pea pot". Day 25: Agro-inoculate pea plants with pea early-browning virus VIGS constructs. Day 37-39: Transfer pea pots to nurse pots. *Day 41-43: In experiments involving nodulation analysis, add *R. leguminosarum *to nurse pot with in-growing pea roots. Day 46-59: Harvest and wash pea roots growing into the nurse pots. Analyze for mycorrhiza colonization, gene expression and nodulation.

The PEBV-VIGS system for silencing studies in *P. sativum *consists of two binary vectors derived from pCAMBIA1300 and named pCAPE1 (HQ687213) and pCAPE2 (HQ687214). The vectors contain the cDNA coding for PEBV RNA1 and PEBV RNA2, respectively [5]. pCAPE2 is modified to allow insertion of a heterologous sequence of the plant gene of interest (GOI) targeted for silencing, using the restriction sites: *XbaI*/*SpeI*/*NcoI *and *PstI*/*BglII*/*EcoRI *flanking the PDS insert in pCAPE2-PDS [[5], Figure 1c].

• Select gene-specific silencing fragment of GOI. Perform siRNA scan to evaluate risk for off-target silencing. It can also be valuable to analyse more than one VIGS fragment per target gene, to ensure that the phenotypes observed are actually caused by specific silencing of the target gene. If targeting a specific member of a gene family consider using the more variable 5'/3' UTR sequences as silencing fragment to minimize the risk for off-target silencing. The size, location and polarity of the inserted fragment can also affect the silencing [3,11,16], for PEBV we recommend fragments of 200-500 bp.

• For the VIGS control construct, select a fragment of corresponding length to the GOI silencing fragment of a control gene, which does not target any endogenous plant genes, for example a virus gene, gus or gfp genes

• Clone the relevant fragments into pCAPE2 using an enzyme in the cloning cassette [5] and transform the resulting VIGS construct into *Agrobacterium tumefaciens *GV3101 by electroporation; plate on selection medium including 100 μg ml^-1 ^rifampicin, 25 μg ml^-1 ^gentamycin and 50 μg ml^-1 ^kanamycin.

Note. We used a Gene Pulser II, (Bio-Rad, Herlev, Denmark) as described by Shen and Forde [17].

• In all experiments, *A. tumefaciens *transformed with pCAPE1 was used in a 1:1 mixture with *A. tumefaciens *transformed with the pCAPE2 construct carrying the GOI silencing fragment.

• Check content of selected *A. tumefaciens *colonies, for example by colony PCR using primers that amplify the GOI.

Note: We chose sequences of approximately 400 bp of *PsSym19*, a gene for which the mutant is characterised as nod^-^myc^- ^[18] and of *PsPT4*, the pea homologue of the mycorrhiza-specific phosphate transporter in *M. truncatula*. We performed siRNA Scan using the siRNA Scan tool: http://bioinfo2.noble.org/rnaiscan.htm with default settings [19] to search for genes with at least 21 nt identical or complementary to the silencing fragment inserted into pCAPE2. Because there is no dataset available for *P. sativum*, siRNA scans were performed against datasets for *M. truncatula *and *L. japonicus*. The siRNA scan identified only sequences of *PsSym19 *and *PsPT4 *homologues, respectively. BLAST searches [20] against the National Centre for Biotechnology Information (NCBI) nucleotide collection identified only homologues of *PsSym19 *and *PsPT4*. As virus control construct we used pCAPE2-Con, which contained a 524 bp insert from the plant virus: bean yellow mosaic virus [5].

### The mycorrhiza-PEBV-VIGS protocol

The mycorrhiza-PEBV-VIGS protocol was developed with the aim of establishing virus infection and generation of siRNA molecules for induction of gene silencing in the pea roots before they became colonized with AMF. This was achieved by allowing the growth of already-silenced roots into a second, nurse pot with an established, active mycelium and subsequent analysis of the new root growth in the nurse pot (Figure 1).

The VIGS constructs were introduced into the pea plants 11 days after sowing by agro-inoculation.

Agro-inoculation:

• Start 3 ml cultures of verified pCAPE2 *A. tumefaciens *clones and of pCAPE1 in Luria broth (LB) supplemented with 100 μg ml^-1 ^rifampicin, 25 μg ml^-1 ^gentamycin and 50 μg ml^-1 ^kanamycin at 28°C for 24 hrs with shaking. Use this culture to start the cultures for plant-infiltration.

• Prepare 2.5 ml LB with 25 μg ml^-1 ^gentamycin and 50 μg ml^-1 ^kanamycin for each plant to be infiltrated. Add 0.3 μl start-culture per ml and incubate at 28°C for 16-18 hrs with shaking.

• Harvest the bacteria at OD550 = 1.2-1.5 by centrifugation (3500 g, room temperature).

• Resuspend cells in infiltration medium (10 mM NaCl, 1.75 mM CaCl_2_, 100 μM acetosyringone), 0.5 ml for each plant to be infiltrated. Incubate at room temperature for 90 min without shaking.

• Infiltrate a 1:1 mix of *A. tumefaciens *cultures carrying pCAPE1 and pCAPE2-GOI respectively to the abaxial side of the youngest pair of leaves using a 1-ml syringe. Use 0.2-1 ml of mixture per plant.

The mycorrhiza-PEBV-VIGS protocol (see Figure 1 for overview):

• (Day 1): Prepare nurse pots by sowing pre-germinated (2 days) *Cucumis sativus *cv 'Aminex' (S&G, the Netherlands) seeds in a bag made from 25 μm nylon mesh http://www.streno.dk containing soil and mycorrhiza inoculum at a 9:1 ratio (w/w). Cucumber roots are retained by the nylon mesh whereas external mycelium proliferates into the surrounding soil volume. Nurse pots are ready as inoculum source 4-5 weeks after sowing.

• (Day 12). Sterilise pea seeds, germinate for 2 days.

• (Day 14). Sow germinated seeds in pots with a perforated bottom, which is sealed at this stage.

• (Day 25). Agro-inoculate pea plants with VIGS constructs.

• (Day 37-39). Transfer pea pots to nurse pots. Remove the shoots and leaves of the cucumber plants growing into the nurse pots; fold the nylon mesh bag containing cucumber roots and cover gently with soil, taking care to minimize disturbance of the mycelium. Remove the bottom perforation seal of the pea pot and place the pea pot directly on the soil contained in the nurse pot. New pea roots will grow into the active mycelium established in the nurse pots and become rapidly AMF-colonized.

• (Day 41-43). In experiments including assays of symbiosis with *R. leguminosarum *bv. viceae strain 248 [12], start the *Rhizobium *culture two days post transfer (dpt) of the pea pots to the nurse pots and apply to the nurse pots as described in methods.

• (Day 46-59). Harvest and analyses of pea roots growing into the nurse pots 9-20 dpt. Pea roots growing into the nurse pot are cut at the base of the pea pot and washed gently, by several rounds of aqueous resuspension and collection of all root pieces on a sieve. Analyze gene expression, symbiotic phenotype(s) and/or P-concentrations in the pea roots.

Note: A good marker for visual inspection of root silencing is still lacking, but from previous studies we know that the photobleaching in leaves resulting from agro-inoculation of plants with pCAPE1/pCAPE2-PsPDS, carrying a fragment of *Phytoene desaturase*, starts to develop approximately ten days post virus inoculation (dpi) [5,11]. We have also observed that the virus has spread to the roots at 8-12 dpi, by *in situ *histochemical assays in plants that were agro-inoculated with pCAPE1/pCAPE2-GUS (data not shown). Based on these observations, transfer of pea pots to nurse pots was timed to the onset of photobleaching in leaves of 10 plants agro-inoculated with pCAPE1/pCAPE2-PsPDS,

### Analysis of roots

#### Quantification of transcript levels

Silencing efficiencies obtained with the described protocol are evaluated by transcript analysis of the VIGS-targeted genes.

• Flash-freeze a sub-sample of total root samples in liquid N_2_.

• Extract total RNA from 50 mg new root material.

Note. We used the RNeasy Plant Mini Kit (Qiagen Hilden, Germany) with on-column DNase treatment following the manufacturer's recommendations. To ensure complete removal of genomic DNA an additional DNase treatment was performed with 0.8 U of RQ1 RNase-free DNase (Promega Corp., Madison, WI, U.S.A.) per 40 μl of RNA eluate for 15 min at 37°C. The DNase reaction was terminated by addition of RQ1 DNase Stop Solution (Promega Corp.) and subsequent incubation for 10 min at 65°C.

• Measure RNA concentrations (we used a Nanodrop ND-1000 Spectrophotometer (Saveen 1 Werner, Malmö, Sweden)).

• Perform first-strand cDNA synthesis with 1 μg of total RNA, with random hexamer primers for the reverse transcriptase (RT) reaction (we used 40 pmol Random Hexamers per sample (PE Applied Biosystems, Forster City, CA, USA) in a total volume of 25 μl according to the manufacturer's protocol (Roche), including the addition of RNase inhibitor.

• Quantify by real-time RT-PCR. Normalize values using real-time RT-PCR data for a relevant gene (18 S rRNA or a housekeeping gene such as EF1a or Ubiquitin).

• Note. We performed Real-time PCR, using the Rotor Gene 2000 Real Time Cycler (Qiagen). RT reactions were diluted 1:3 in H_2_O. Each 25 μl of PCR reaction contained 2 μl of diluted RT reaction, 12.5 μl of Maxima SYBR Green qPCR Master Mix (Fermentas) and 500 nM of each primer. Samples were heated to 95°C for 10 min, followed by 40 cycles of 15 sec at 95°C and 1 min at 60°C. The Rotor Gene 2000 software calculated relative amounts of RNA based on Ct values obtained from a dilution series from 2,5^-1^-6,25^-3 ^(each step a 1:3 dilution in H_2_O) of a standard RT sample from a plant inoculated with pCAPE1/pCAPE2-Con. Each RT reaction was analysed in duplicate. Calculated relative amounts of target gene RNA were normalized by calculated relative amounts of 18 S rRNA.

#### Symbiotic phenotypes of plants agro-inoculated with VIGS constructs

• In experiments where nodulation studies are included: Evaluate nodule formation of the whole root system before sampling for other analyses. Visible nodules are counted and grouped into white (young nodules) and pink (still developing and mature) nodules if this is relevant.

• For AMF-colonization analysis. Harvest a sub-sample of total root sample from the nurse pot, clear with 10% KOH and stain with either trypan blue [21], omitting phenol from the reagent and HCl from the rinse, or Schaeffer black ink [22]. Determine root length as described by Newman [23] and assess the proportion of root length with mycorrhizal structures (arbuscules and/or vesicles) by recording presence or absence of structure at each intersection between roots and hairline in microscope eyepiece.

• Note. We have tested whether inoculation with the PEBV-VIGS vector would interfere with mycorrhiza colonization in pea roots. AMF colonization of plants agro-inoculated with pCAPE1/pCAPE2-Con was compared to AMF colonization of plants exposed to buffer only. Roots growing into the *G. intraradices *nurse pots during a 19-day exposure period had 90% of the root length colonized, irrespective of whether plants were previously infected with PEBV (82 - 100%) or mock-inoculated (86 - 93%).

#### Phosphate uptake analysis in plants agro-inoculated with VIGS constructs

• Dry shoots at 80°C for 48 hrs.

• Crush the roots which were flash-frozen in liquid N_2 _at harvest. Keep two aliquots of 50 mg root tissue from each plant at -80°C for RNA isolation (see above). Determine wet weight and dry the rest of the root tissue at 80°C for 48 hrs.

• Determine dry weights for shoot and root samples.

• Digest ground and homogenized dry tissue samples in 4:1 65% nitric-70% perchloric acid mixture to convert all P compounds into phosphate (Pi). Determine Pi concentration by the molybdate blue method [24] using AutoAnalyzer 3 (Bran+Luebbe, Norderstedt, Germany).

## Comments

The protocol was used to silence *PsSym19 *and *PsPT4*, two genes that are important early and late during AMF symbiosis and we obtained effective and reproducible silencing. Approximately 400 bp silencing fragments of each gene were inserted into pCAPE2. Total RNA was extracted from 100 mg nodulated or AMF-colonized root tissue of *P. sativum *cv. 'Dark Skinned Perfection' in order to amplify cDNA fragments of *PsSym19 *and *PsPT4*. One μg RNA was used for first-strand cDNA synthesis with oligo dT primers for the reverse transcriptase (RT) reaction. The silencing construct pCAPE2-PsSym19 was generated by PCR amplification of a 411 bp DNA fragment of *PsSym19*, corresponding to nucleotides 649 to 1059 of AF491997 [25], using forward primer 5'- ccatggcGTTTCGATTGGG GCAACAGAAC and reverse primer 5'- gaattcGACGAACTCCAATCGCTCAG. The product was inserted into pCAPE2-PDS as a *Nco*I/*Eco*RI fragment. Accordingly, pCAPE2-PsPT4 was generated by PCR amplification of a 427 bp cDNA fragment of *PsPT4*, corresponding to nucleotides 618 to 1044 of GU721059 using forward primer 5'- cctctagaGGTGATTTATTATGGCGTCTAGTTCTCATG and reverse primer 5'- cgagatctCCAAACAATGCAATGATGAACATGGCAC. The product was inserted into pCAPE2-PDS as a *XbaI*/*BglII *fragment. All fragments were cloned using TOPO13 technology (Invitrogen, Taastrup, Denmark) and verified by sequencing.

Transcripts of *PsSym19 *and *PsPT4 *were quantified by real-time RT-PCR and normalized by expression data for 18 S rRNA, (see Table 1 for primers). Silencing of *PsSym19 *resulted in decreases in *PsSym19 *transcript levels in all the root samples analysed in a range from 35%-68% with averages of 51% and 58% in two separate experiments, a and b, (Table 2). In the case of *PsPT4*, transcript levels were reduced in 19 of the 20 root samples analysed in a range from from 30%-72% with an average of 52% at 20 dpt in one experiment (c) and averages 60% and 47% at 14 and 20 dpt, respectively, in a second experiment (d) (Table 3). These data show that agro-inoculation of *P. sativum *with pCAPE2-PsSym19 or pCAPE2-PsPT4 caused a significant and reproducible reduction in the respective transcripts of the target genes in AMF colonized roots.

**Table 1:** Primers used for quantitative real time PCR.

Target	**Primer sequences (designed using Primer 3 **[26])
*18S*: U43011	Fw: 5'-GACTACGTCCCTGCCCTTTG (nt 1571-1590) Rev: 5'- AACACTTCACCGGACCATTCA (nt 1618-1638)

*PsSym19*: AF491997	Fw: 5'- GATTGGTTGTGGCGTGTGTTC (nt 317-337) Rev: 5'- GCTCTCAAACCCTTCAGTTGC (nt 373-393)

*PsPT4*: GU721059	Fw:5'-CAATATTGTCACCGGTGTGGCACTTGTC (nt 197-224) Rev: 5' GGAAGAACCGAATGAAAGGCCGGAGCA (nt 327-353)

**Table 2:** Expression levels of PsSym19 in virus control plants and silenced plants

Experiment	Relative transcript levels of *PsSym19*	
	pCAPE2-Con	pCAPE2-PsSym19
a, 20 dpt	100 ± 41	49 ± 10
b, 20 dpt	100 ± 14	42 ± 5

Numbers are means of relative transcript levels ± 95% confidence intervals.

**Table 3:** Expression levels of PsPT4 in virus control plants and silenced plants

Experiment	Relative transcript levels of *PsPT4*	
	pCAPE2-Con	pCAPE2-PsPT4
c, 20 dpt	100 ± 16	48 ± 12
d, 14 dpt	100 ± 20	40 ± 7
d, 20 dpt	100 ± 22	53 ± 11

Numbers are means of relative transcript levels ± 95% confidence intervals.

**Figure 2:**
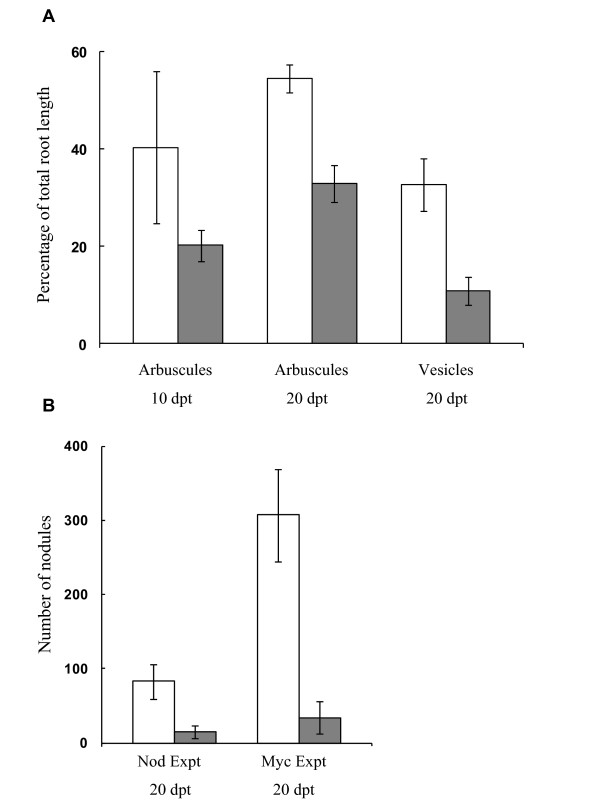
**Mycorrhiza colonization and nodule numbers in roots of *Pisum sativum *inoculated with pCAPE2-PsSym19**. (A) Percentage of total root length with arbuscules 10 and 20 days post transfer (dpt) and vesicles 20 dpt in plants agro-inoculated with pCAPE2-Con (control, white columns) or pCAPE2-PsSym19 (Sym19, grey columns). Results are presented for one of two experiments showing similar results. (B) Number of visible nodules per plant 20 dpt. Plants were agro-inoculated with pCAPE2-Con (control, white columns) or pCAPE2-PsSym19 (Sym19, grey columns). Plants were grown in the mycorrhiza-PEBV-VIGS set-up "Myc Exp" (described in this paper) or in the nodulation -VIGS setup "Nod Exp" [5]. Columns and bars are means ± 95% confidence intervals (CI). VIGS effect was significant for all measured variables.

In experiment (b), targeting *PsSym19 *for silencing, AMF colonization and nodulation was evaluated in new pea roots growing into the nurse pot. Development of both arbuscules and vesicles was significant less in plants agro-inoculated with pCAPE2-PsSym19 than in plants agro-inoculated with pCAPE2-Con (Figure 2A). Silencing of *PsSym19 *resulted in a reduction in the proportion of root length with arbuscules being 50% at 10 dpt and 40% at 20 dpt. No vesicles were formed 10 dpt in any of the plants, but the proportion of root length with vesicles was reduced by 67% at 20 dpt. Nodule development was also inhibited by *PsSym19 *silencing. The total number of visible nodules was evaluated 20 dpt and in plants agro-inoculated with pCAPE2-PsSym19 the nodule number was reduced by 89% compared to plants agro-inoculated with pCAPE2-Con (Figure 2B).

**Figure 3:**
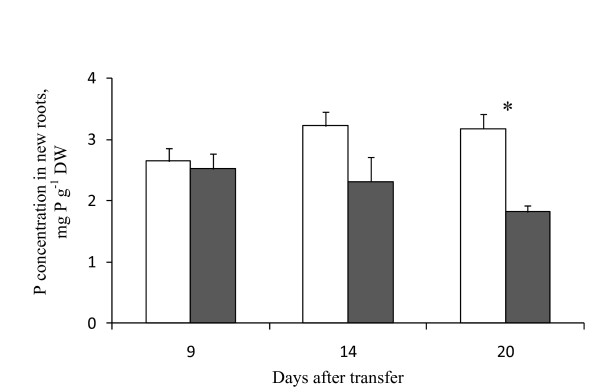
**Phosphorus (P) concentration in roots of *Pisum sativum *agro-inoculated with pCAPE2-PsPT4**. Phosphorus concentration in pea roots growing into the nurse pots 9, 14 and 20 dpt. Plants had been agro-inoculated with pCAPE2-Con (white columns) or pCAPE2-PsPT4 (grey columns). Data are means ± 95% CI. *Significant difference between treatments.

In experiment (d), targeting *PsPT4 *for silencing resulted in a reduced capacity of the new roots to take up phosphate. The phosphorus concentration in new roots, growing into the nurse pot, from plants that were agro-inoculated with pCAPE2-PsPT4 was reduced significantly by an average of 42% (*P *< 0.0006) compared to plants agro-inoculated with pCAPE-Con at 20 dpt. This effect increased over time but was not significant at 9 and 14 dpt (Figure 3).

## Conclusions

The mycorrhiza-PEBV-VIGS protocol developed in this work proved successful in obtaining pea plants silenced for *PsSym19 *and *PsPT4*, which displayed altered symbiotic behaviour concerning AMF colonization and phosphate uptake, respectively. The advantage of the protocol is that roots produced after implementation of gene silencing grow directly into the nurse pot, which contains an already established AMF mycelium. In consequence, these roots become rapidly colonized and during harvest they are easily separated from roots in the upper pea pot, which were not significantly silenced (data not shown) probably because a large proportion of these roots had developed before virus inoculation. The protocol can therefore be used as an alternative reverse genetics tool for silencing of genes involved in both early and late events in the AMF symbiosis. The protocol can also be adapted for VIGS systems other than PEBV-VIGS for functional studies of genes involved in the AMF-symbiosis and for studies of other root infecting organisms.

## Competing interests

The authors declare that they have no competing interests.

## Authors' contributions

MG and IJ conceived and designed the study, MG carried out the molecular studies and all analyses described and drafted the manuscript. AO participated in the AMF and P analysis and EJ provided the expertise in VIGS and performed the statistical analysis. All authors read and approved the final manuscript.
